# Targeting TOR and SnRK1 Genes in Rice with CRISPR/Cas9

**DOI:** 10.3390/plants11111453

**Published:** 2022-05-30

**Authors:** Bhuvan Pathak, Chandan Maurya, Maria C. Faria, Zahra Alizada, Soumen Nandy, Shan Zhao, Muhammed Jamsheer K, Vibha Srivastava

**Affiliations:** 1 Department of Crop, Soil & Environmental Sciences, University of Arkansas System Division of Agriculture, Fayetteville, AR 72701, USA; bhuvan.pathak@ahduni.edu.in (B.P.); maurya@uark.edu (C.M.); mcfariac@uark.edu (M.C.F.); snandy@uark.edu (S.N.); shanzhao@uark.edu (S.Z.); 2 Cell and Molecular Biology Program, University of Arkansas, Fayetteville, AR 72701, USA; zahraalizada2019@gmail.com; 3 Amity Institute of Genome Engineering, Amity University Uttar Pradesh, Noida 201313, India; muhdjamsheer@gmail.com

**Keywords:** tor, SnRK1, CRISPR/Cas9, targeted mutagenesis, essential genes, rice

## Abstract

Genome targeting with CRISPR/Cas9 is a popular method for introducing mutations and creating knock-out effects. However, limited information is currently available on the mutagenesis of essential genes. This study investigated the efficiency of CRISPR/Cas9 in targeting rice essential genes: the singleton *TARGET OF RAPAMYCIN* (*OsTOR*) and the three paralogs of the Sucrose non-fermenting-1 (SNF1)-related kinase 1 (*OsSnRK1α*), *OsSnRK1αA, OsSnRK1αB* and *OsSnRK1αC.* Strong activity of constitutively expressed CRISPR/Cas9 was effective in creating mutations in *OsTOR* and *OsSnRK1α* genes, but inducible CRISPR/Cas9 failed to generate detectable mutations. The rate of *OsTOR* mutagenesis was relatively lower and only the kinase domain of *OsTOR* could be targeted, while mutations in the HEAT region were unrecoverable. *OsSnRK1α* paralogs could be targeted at higher rates; however, sterility or early senescence was observed in >50% of the primary mutants. Additionally, *OsSnRK1αB* and *OsSnRK1αC*, which bear high sequence homologies, could be targeted simultaneously to generate double-mutants. Further, although limited types of mutations were found in the surviving mutants, the recovered lines displayed loss-of-function or knockdown *tor* or *snrk1* phenotypes. Overall, our data show that mutations in these essential genes can be created by CRISPR/Cas9 to facilitate investigations on their roles in plant development and environmental response in rice.

## 1. Introduction

Targeted mutagenesis is foundational in deciphering gene functions and the associated genetic pathways. The advent of CRISPR/Cas9 has paved the way for targeted mutagenesis in a wide range of plant species. The simple rule of CRISPR/Cas9-targeting that involves the formation of a double-stranded break (DSB) in the sequences ending with the NGG motif makes virtually all genes a candidate for mutagenesis. The DSB is repaired by the host’s non-homologous end-joining (NHEJ) pathways, resulting in insertion-deletions (indels) at the DSB site, precisely 3 bp upstream of NGG [1,2,3]. The incorporation of indels in the genic sequences often leads to the knock-out effect through frame-shift and the incorporation of an early stop codon. Numerous studies have demonstrated high rates of targeting, resulting in ±1 bp or short indels at the DSB sites [4,5,6]. The resulting mutants are critical for linking genotypes with phenotypes. The mutants could also display useful traits and serve as breeding lines for crop improvement [7,8,9]. However, the viability and fertility of mutants are the key to their application in functional genomics or biotechnology.

Mutations in essential genes are deselected, as their disruption may lead to severe growth defects or lethality. In the absence of mutants, approaches for deciphering gene functions are somewhat limited. Full sets of essential genes are currently unknown for any plant species and many likely essential genes have no assigned phenotypes [10,11,12]. CRISPR/Cas9 mediated mutagenesis could be used to confirm the status of essential genes or reveal new essential genes [13], but the question remains whether the resulting mutants will survive and do the survivors carry a silent or knockdown mutation? The viability of the mutants carrying a knock-out effect in the essential gene, or the gene copy, will depend on the function of the gene in development and/or reproduction. A vast majority of such mutants are presumably unrecoverable; however, knockdown or conditional mutations are possibly recoverable and heritable.

Rates of natural or induced (random) mutations in essential genes are greatly reduced or masked by lethality [14]. Arguably, the extraordinary efficiency of CRISPR/Cas9 could be leveraged to generate a variety of mutations in essential genes that might help in establishing genotype–phenotype linkage. It is, therefore, worth investigating whether CRISPR/Cas9-targeting could increase the rate of mutagenesis in essential genes and facilitate the recovery of stable lines. Alternatively, could essential genes be targeted by inducible CRISPR/Cas9 to generate mutations in a specific tissue or developmental phase and avoid lethality or sterility in the plant?

In this study, two conserved protein kinases, TOR and SnRK1, were targeted by CRISPR/Cas9 in rice. TOR and SnRK1 complexes are central regulators of metabolism under nutrient-rich or starvation conditions, respectively, that play important roles in plant growth and development [15,16,17]. Rice possesses a single copy of the *TOR* gene [18,19]. On the other hand, three paralogs of SnRK1 kinase subunit (aka α subunits) genes (A, B, and C) are found in rice [18,19], and recently, another highly diverged paralog of SnRK1α (D) was identified [20].

Consistent with the understanding that mutations in singleton essential genes are not selected, mutations in *OsTOR* were found in only 14% of plant lines transformed with the CRISPR/Cas9 vector. Among the recovered *OsTOR* mutants, one contained a 1 bp insertion (+1) at the target site and the remaining two contained in-frame monoallelic or biallelic deletions (Δ) of 3, 6 or 9 bp. Of these, +1 was not heritable, indicating the critical function of TOR in rice gamete and/or embryo development. The Δ6 could not be studied as the plant failed to set seeds. Most importantly, homozygotes of Δ3 and Δ9 were healthy and fertile and showed a knockdown phenotype. Mutations in *OsSnRK1α* genes, on the other hand, could be recovered at higher rates, but sterility or severe growth arrest were frequently observed among the mutants. Interestingly, biallelic homozygous mutations in *SnRK1α* genes were a common occurrence, and *OsSnRK1αB* and *OsSnRK1αC* paralogs could be targeted concomitantly at a high rate. A higher rate of plant death after reaching the mid-to-late vegetative stage indicates the “essentiality” of *OsSnRK1α* paralogs. Further, *OsSnRK1αB* and *OsSnRK1αC* could be targeted simultaneously, however, at least one functional allele of either gene appeared to be necessary for the recovery of the mutant, indicating functional redundancy and dose effect of these genes. Homozygous mutations in *OsSnRK1αA*, on the other hand, were readily obtained, however, they were associated with lower seed sets in the mutant plants. Finally, attempts to generate somatic mutations in *OsTOR* by inducible CRISPR/Cas9 systems, that were efficacious on non-essential genes [4], were unsuccessful.

Overall, depending on the essentiality of the gene or gene copy, strong or weak mutations can be generated by CRISPR/Cas9, some of which are heritable. The established *OsTOR* or *OsSnRK1α* mutants serve as unique resources for deciphering signaling pathways underlying the central metabolic processes and investigating their specialized function in the plant.

## 2. Results

### 2.1. OsTOR Targeting

**Figure 1:**
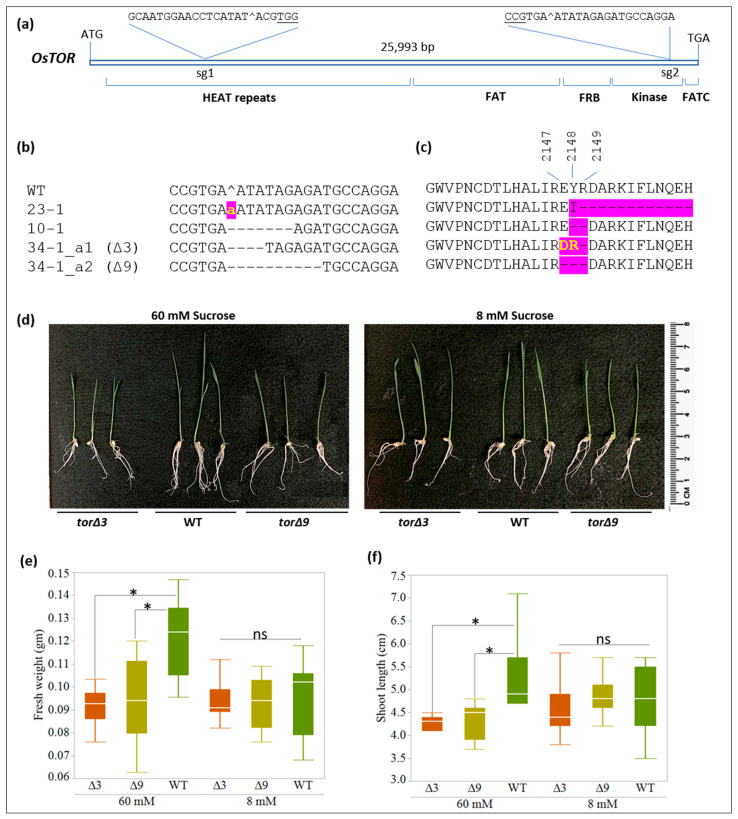
Targeting of *OsTOR* by CRISPR/Cas9. (**a**) Structure of *OsTOR* showing sgRNA targeting sites (sg1 and sg2) in the HEAT region and kinase domain sequences. Protospacer adjacent motif (PAM) is underlined and the predicted double-stranded break (DSB) site is indicated (^). (**b**) Alignment of the targeted *OsTOR* sequences of the mutant lines and the wild-type (WT). Yellow small letter indicates insertion and dashed line show deletions. (**c**) Predicted TOR protein sequences showing changes in the highlighted area. The amino acids affected by targeting (2147–2149) are indicated. Yellow large letters indicate substitutions and dashed lines show deletions. (**d**) Representative 9-d-old os*tor* (harboring Δ3 or Δ9 mutations) and WT seedlings grown in MS½ media supplemented with 60 mM or 8 mM sucrose. (**e**,**f**) Shoot length and fresh weight of WT and os*tor* mutants measured in 9-d-old seedlings grown in 60 mM or 8 mM sucrose. Statistical differences (*p* < 0.05, *n* = 10) by Tukey–Kramer test (HSD) are shown as significant (*) or non-significant (ns).

TOR is a highly conserved Se-Thr kinase that consists of HEAT (*h*untingtin, *e*longation factor 3, *a* subunit of PP2A, and *T*OR1) repeats at the N-terminal and the catalytic kinase domain at the C-terminal among other domains that participate in the formation of TOR complex 1 (TORC1). The HEAT region is important for protein–protein interaction and works as a conserved site of interaction with accessory subunit RAPTOR [19,21]. We targeted the *OsTOR* (LOC_Os05g14550) HEAT region and the kinase domain by a dual-targeting CRISPR/Cas9 construct, pNS71, that targeted the 9th and 50th exon of *OsTOR* (Figure 1a).

None of the 21 CRISPR-transformed lines (T0) showed mutations in the HEAT region, whereas three lines showed targeted mutations in the kinase domain disrupting the conserved EYR sequence at 2147–2149 position and/or the subsequent sequence in the protein (Figure 1b,c; Table 1). These residues belong to a highly conserved region in the plant, yeast and mammalian TOR kinases (Figure S1), and the structural analysis reveals that this region is very close to the core helix–loop–helix LST8-binding site of TOR [22]. LST8 is an essential component of TORC1 [23]. The R residue is highly conserved in all eukaryotic (fungi, animal and plant) TOR kinases, and Y is fairly conserved in plant TOR kinases (Figure S1). T0_10-1 contained a monoallelic in-frame 6 bp deletion (Δ6) at the target site that resulted in the deletion of YR in the conserved sequence. However, since T0_10-1 was not fertile, the inheritance of ΔYR could not be studied. T0_23-1 contained a monoallelic 1 bp insertion (+1) at the target site, resulting in an early stop codon and truncation of the TOR reading frame (Figure 1b,c). Analysis of T0_23-1 progeny revealed that +1 mutation was not heritable as only the WT allele was found in >22 T1 lines sequenced at the target sites (data not shown). These results agree with previous reports of embryo lethality in *tor* null mutants of mice and Arabidopsis [24,25].

Finally, T0_34-1 was found to contain biallelic heterozygous mutations in the target site, consisting of in-frame 3 or 9 bp deletions (Δ3, Δ9) in the two alleles (34-1_a1 and 34-1_a2) (Figure 1b). The Δ3 mutation led to E to D and Y to R substitutions and R deletion at the EYR conserved site, and the Δ9 mutation generated an in-frame EYR deletion in the kinase domain (Figure 1c). Each mutation was transmitted independently to the progeny and all T1 plants, including those homozygous for Δ3 and Δ9, were healthy and fertile. To determine whether these mutants show any defect in TOR signaling, seedlings were grown in the media containing either 8 mM or 60 mM sucrose, representing low and high energy environments, respectively. As a source of energy, sucrose is known to promote the growth of the seedlings germinated in vitro and this growth is mediated by TOR signaling [26,27]. However, this response is subdued under a low-energy environment. Accordingly, shoot length and seedling biomass (fresh weight) of the two *ostor* mutant lines were lower than that of WT in 60 mM sucrose, while no significant differences were observed among seedlings grown on 8 mM sucrose (Figure 1d–f).

In summary, *OsTOR* could be targeted at a low frequency by CRISPR/Cas9 (Table 1). While null *tor* mutations were non-heritable, point-mutations leading to in-frame substitution and/or deletions were established that potentially have a knockdown effect on TOR kinase activity. Notably, deletion of R-2149, which is highly conserved across genera and species (Figure S1) presumably subdued TOR signaling, as evident by reduced seedling growth under energy-sufficiency conditions. This study also indicates the importance of the HEAT region in the formation of functional TORC1, as this region was refractory to accumulating mutations. Interestingly, plants harboring a heterozygous null mutation in the kinase domain (T0_23-1) were healthy through the vegetative phase, indicating the sufficiency of one working copy of *OsTOR* (dosage-effect) during vegetative growth and development. However, as reported for mammals and Arabidopsis [24,25], the null *tor* mutation in rice was not transmitted to the next generation, presumably due to embryo lethality.

### 2.2. OsSnRK1α Targeting

**Figure 2:**
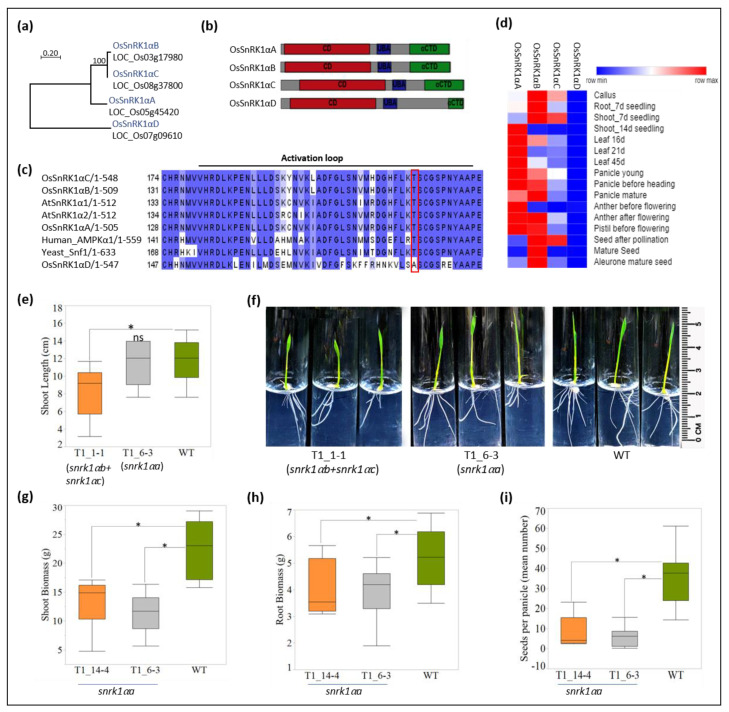
Targeting of *OsSnRK1* kinase subunits (α-subunits) genes. (**a**) Neighbor joining-based phylogenetic reconstruction of rice SnRK1α subunits. (**b**) Domain organization of rice SnRK1α subunits. The domain organization was analyzed by CDD v3.19. CD: catalytic domain; UBA: ubiquitin-associated domain; αCTD: αC-terminal domain. (**c**) Sequence alignment of rice SnRK1α subunits with Arabidopsis, yeast and human homologs. The sequences were aligned with ClustalX2 and visualized in Jalview (v2.11.2.0). (**d**) Expression level (FPKM) of rice *SnRK1α* subunits in different tissue and developmental stages. The expression data were retrieved from Rice Expression Database [31]. (**e**–**f**) Seedling length of 7-d-old *snrk1*α double or single mutants and the WT grown in MS½ media (**e**) and representative images of the same (**f**). (**g**–**i**) Shoot biomass, root biomass, and mean number of seeds per panicle in *snrk1*αa mutant lines in comparison to WT. Statistical differences (*p* < 0.05, *n* = 10) by Tukey–Kramer test (HSD) are shown as significant (*) or non-significant (ns).

SnRK1 is an evolutionarily conserved kinase and plants possess both catalytic α (kinase) and regulatory β and βγ subunits [15,28]. However, a recent study in Arabidopsis identified that plant SnRK1α are regulatory subunit-independent, constitutively active kinases [29]. Phylogenetic reconstruction of rice OsSnRK1α protein sequences reveals that OsSnRK1αD is a highly diverged paralog, while OsSnRK1αB and C show very high similarities (Figure 2a). All paralogs show the canonical domain composition with the N-terminal catalytic domain (CD) followed by the ubiquitin-associated domain (UBA) and αC-terminal domain (CTD) (Figure 2b; [15,28]). Strikingly, the critical T residue in the activation loop important for the kinase activity of SnRK1 [30], was found to be replaced by an A residue in OsSnRK1αD (Figure 2c). The spatiotemporal expression analysis revealed that, in comparison to other paralogs, *OsSnRK1αD* is feebly expressed (Figure 2d). Considering the mutation in the T-loop and low expression, it seems that *OsSnRK1αD* has a limited contribution to the SnRK1 activity in plants. Therefore, we selected the other three paralogs (*OsSnRK1αA-C*) for targeting SnRK1 activity in rice in two separate experiments. Due to high sequence similarity, *OsSnRK1αB* and *OsSnRK1αC* were targeted in the kinase domain by a single CRISPR/Cas9 construct, pNS72, and the two targeted sites (sg1 and sg2 sites) bear 100% sequence homology between *OsSnRK1αB* and *OsSnRK1αC* genes (Figure S2a). *OsSnRK1αA*, on the other hand, was targeted by pNS73 at two different target sites in the kinase domain (Figure S2b).

Relatively low transformation efficiency was observed with pNS72, generating a total of 10 T0 plants, 9 of which harbored monoallelic or biallelic mutations in one or both targeted sites. Four of these lines did not grow well, failed to set seeds, and died prematurely (Table 1). Sterility among tissue-culture-derived rice is not uncommon, however, severe growth retardation and premature senescence are likely associated with *snrk1αb/snrk1αc* mutations. To differentiate mutations in *OsSnRK1αB* and *OsSnRK1αC* in the surviving lines, PCR was done with gene-specific primers followed by amplicon sequencing.

The analysis of sequences showed that T0_1-1, T0_4-1, and T0_4-2 had identical mutations in both *OsSnRK1αB* and *OsSnRK1αC*, indicating their possible clonal origin. They are referred to as T0_1-1, hereafter. Notably, all lines contained mutations in both genes; however, the sg2 site in both genes was targeted more often, indicating higher efficiency of sgRNA2. T0_1-1 and T0_1-2 contained identical biallelic heterozygous mutations in *OsSnRK1αB*, resulting in an early stop codon in allele 1 and 1 amino acid (aa) deletion in allele 2 (Figure S2a). However, the two lines differed in the targeting of *OsSnRK1αC*. T0_1-1 contained biallelic (homozygous) 5 bp deletion (Δ5), while T0_1-2 contained Δ5 in allele 1 and T to G substitution in allele 2, resulting in an early stop codon and 1 aa substitution, respectively (Figure S2a). T0_2-4 contained the biallelic homozygous 69 bp insertion (+69) in *OsSnRK1αB* and biallelic heterozygous mutations in *OsSnRK1αC*, resulting in an early stop codon in allele 1 and 1 aa deletion in allele 2 (Figure S2a). A search of the sequence homology using BLAST showed that the +69 sequence bears 100% homology to rice mitochondrial petB pseudogene (GenBank: D13104.1). In summary, we recovered three surviving lines (T0_1-1, T0_1-2, and T0_2-4) harboring monoallelic or biallelic mutations in the kinase domains of *OsSnRK1αB* and *OsSnRK1αC* that generated an early stop in the reading frame or in-frame deletion. Both target sites are located prior to the kinase active site, and the presence of early stop codon in both alleles potentially obliterates kinase activity. Two of these lines (T0_1-1 and T0_2-4) harbored biallelic homozygous mutations, resulting in an early stop codon in *OsSnRK1αB* or *OsSnRK1αC,* however, not in both genes at the same time. Since *OsSnRK1αB* and *OsSnRK1αC* are highly expressed in callus and early vegetative stages, one functional copy of either gene is possibly required for the recovery of mutants through tissue culture and regeneration. However, double-homozygous null lines (*snrk1αb/snrk1αb*:*snrk1αc/snrk1αc*) could be recovered in the T1 generation (data not shown). Therefore, null mutations in these genes are heritable.

Next, *OsSnRK1αA* was targeted at two sites by pNS73. The resulting 40 T0 lines were analyzed by PCR and amplicon sequencing. PCR across the two target sites in a subset of these lines at a young vegetative stage indicated the presence of somatic deletions (Figure S3). Further, amplicon sequencing of the DNA extracted from mature plants showed point mutations in 30 T0 lines, while the remaining 10 contained wild-type sequences at the two target sites. Thus, *OsSnRK1αA* could be targeted at a higher rate. However, weak architecture and poor growth were commonly observed among the targeted lines, several of which (20 lines) were lost to severe growth defects and premature senescence (Table 1).

Of the surviving 10 lines, 9 contained biallelic homozygous or heterozygous mutations at both target sites, while a single line (T0_13-1) contained monoallelic mutations (Figure S2b). All lines, except T0_13-1, showed a high rate of sterility and produced only 2–5 seeds per panicle. Overall, only three types of mutations were observed among these lines. Seven lines contained 1 bp deletion (Δ1) at sg1 and sg2, six of which were homozygous for each mutation. One line (T0_10-1) contained biallelic homozygous Δ3 at sg1 and Δ11 at sg2, and the remaining two lines (T0_14-4, T0_14-5) contained biallelic Δ1 and +1 at sg1 and ±1 and Δ1 at sg2. All of these mutations, except Δ3 at sg1, resulted in frame-shift and early stop. However, since both sg1 and sg2 were targeted in each line, early stop was incorporated in each line (Figure S2b).

In summary, *OsSnRK1αA* could be targeted at a higher rate, but targeted lines frequently succumbed to severe growth defects and premature senescence. Among the surviving lines, a low rate of seed set was observed in all showing biallelic mutations, indicating an important function of *OsSnRK1αA* in the reproductive phase. In line with this, *OsSnRK1αA* is highly expressed in panicle and reproductive organs (Figure 2d). The observed mutations were heritable and the homozygous mutant lines showed growth retardation and low fertility (see below), possibly due to defects in SnRK1 signaling.

### 2.3. Phenotyping Snrk1 Mutants

SnRK1 is very important for survival under low-energy conditions [30,32]. Recent studies suggest that SnRK1 also plays an important role in normal growth and development [20,28]. Accordingly, the *snrk1αb+snrk1αc* double mutant line T0_1-1 and *snrk1αa* single mutant line T0_6-3 were compared with WT through early stages of seedling growth upon exposure to a period of darkness in media lacking sucrose (mimicking starvation). The shoot length of T1 progeny (7 days after germination) derived from T0_1-1 was significantly lower than compared to that of WT, whereas no difference was observed between T1 seedlings of T0_6-3 and WT (Figure 2e,f). However, both mutant lines showed reduced root growth in the media when compared to the WT (Figure 2f). Thus, early vegetative growth under low-energy conditions is more suppressed in the *snrk1αb+snrk1αc* double mutant. In line with these observations, *OsSnRK1αB* and *OsSnRK1αC* are highly expressed in the early seedling stages (Figure 2d). Overall, these results indicate the potential functional specialization of *OsSnRK1α* paralogs.

To further clarify the phenotype of *snrk1αa* mutants, T1 plants of T0_6-3 and T0_14-4 along with WT were grown in the greenhouse until maturity (119 days after planting). Analyses of the shoot and root biomass of mature plants showed a significant difference between the mutants and the WT. Both *snrk1α*a mutant lines showed reduced shoot and root biomass and a number of seeds per panicle (Figure 2g–i). Hence, *snrk1αa* mutants were compromised in yield components as compared to the WT. These results corroborate recent reports of reduced plant height and reduced seed set in Os*SnRK1αA* knock-out plants grown in normal conditions [20,33].

### 2.4. Testing the Efficacy of Inducible CRISPR/Cas9 on OsTOR

A practical approach to circumventing embryo lethality associated with mutations is to induce mutagenesis during vegetative development. Earlier, we showed that the heat-shock (HS) inducible CRISPR/Cas9 system was effective in targeted mutagenesis in rice [4]. To determine if *OsTOR* could be targeted by inducible CRISPR/Cas9, we cloned the sgRNA cassette into a separate vector and co-bombarded it with heat-inducible (HS:Cas9) or cold-inducible (RD29a:Cas9) Cas9 expression vector as described earlier [4]. A total of 40 T0 plants were developed, however, none of them showed mutations in *OsTOR* at room temperature or after heat or cold treatments of the seedlings (data not shown). Inducible mutations could be difficult to detect in a complex organism. Therefore, we selected six lines based on the highly inducible expression of Cas9 and screened >100 progeny in bulk. However, none of the progeny showed mutations in *OsTOR* sg2 site, which was found to be targeted by strong constitutive Cas9 as described above. These results indicate that mutagenesis of singleton essential genes, such as *OsTOR*, is highly suppressed and a strong dosage of sgRNA/Cas9 is needed for targeting them.

## 3. Discussion

The establishment of mutants greatly facilitates gene function studies. However, mutations in essential genes are suppressed or masked by embryo lethality or growth arrest. Here, we addressed whether CRISPR/Cas9 could generate viable mutations in the singleton *OsTOR* and the three paralogs of *OsSnRK1α*. As expected, mutagenesis of *OsTOR* was suppressed, yet two fertile mutant lines were recovered that harbored 3 bp or 9 bp deletion (Δ), resulting in substitution and in-frame deletion of one or three amino acids. Most importantly, these mutants displayed a knockdown phenotype as indicated by reduced shoot and root length and the seedling biomass.

Disruption of TOR signaling by chemical treatment or RNAi has been linked with a reduced shoot and root biomass, and root length in rice and Arabidopsis [18,27,34]. In fact, *TOR* expression affects phenotype in a dose-dependent manner as modest *TOR* overexpression and RNAi in Arabidopsis led to an increase or decrease in organ size or biomass [34]. Attempts to create overexpression lines reveal that Arabidopsis *TOR* is a highly dosage-sensitive gene and an optimal TOR level is required for normal development [35]. Interestingly, Δ3 *ostor* seedlings showed greater phenotypic defect as compared to Δ9 *ostor* seedlings, which could be based on the combined effect of amino acid substitution and deletion (Figure 1c). Future studies will be focused on assessing the impact of these mutations on TORC1 function using biochemical and kinase activity assays. The establishment of these mutants could enable studies on understanding the function of *OsTOR* in plant development and environmental response. These mutants could also serve as useful tools for deciphering the role of TOR signaling in balancing growth and stress responses, including biotic stress. Chemical or RNAi inhibition of TOR was linked with higher resistance to *Fusarium graminearum* in Arabidopsis [36]. Similarly, in rice, suppression of TOR signaling was linked with higher resistance to leaf blight caused by *Xanthomonas oryzae* pv. *Oryzae;* whereas TOR overexpression correlated with greater susceptibility to the pathogen [18]. The *ostor* mutants are therefore excellent candidates for testing disease. Overall, this study demonstrates that unique variants of essential genes could be generated by CRISPR/Cas9.

SnRK1 signaling is at the crux of metabolic regulation during starvation [30,37]. However, the functions of SnRK1 signaling in plant growth and environmental response are not fully understood. Nonetheless, interactome and phosphoproteome analysis reveal that SnRK1 is a hub protein complex that communicates with diverse core cellular pathways [15]. The development of stable mutant lines is important in enabling genomic, genetic and biochemical experiments toward understanding SnRK1 functions. In this study, *OsSnRK1αB* and *OsSnRK1αC* which bear 81% sequence homology were targeted by a single construct. Simultaneous targeting of paralogous genes is reportedly highly variable, possibly due to the variable efficiency of sgRNAs on the paralogs [38]. However, with efficient sgRNAs, homozygous mutations in paralogous genes are commonly found [39]. We observed simultaneous targeting of *OsSnRK1αB* and *OsSnRK1αC* in every recovered line, indicating the high efficiency of our designed sgRNAs. Therefore, the low transformation rate observed in SnRK1αB + SnRK1αC targeting could be based on the defects in double-homozygous mutations, which points to the essentiality of these genes in rice. SnRK1 mutants have been described earlier. Lu et al. [37] isolated T-DNA insertion lines of rice SnRK1αA_ LOC_Os05g45420 and SnRK1αC_ LOC_Os08g37800 and found that germination and seedling growth are impacted in *snrk1αa* but not in *snrk1αc* mutant. More recently, rice mutants in *SnRK1αA* were developed by CRISPR/Cas9 that were instrumental in deciphering the regulation of sugar homeostasis under normal conditions or starvation and translocation of starch from sheath to panicle during grain filling in rice [20,33]. However, this is the first time, to our knowledge, *snrk1αb + snrk1αc* double-mutants are reported. The similarity in their expression patterns points to their functional redundancy; the use of double-mutants could lead to a better understanding of SnRK1 signaling.

In conclusion, novel variants of essential genes could be generated by CRISPR/Cas9. These mutants could serve as important tools for deciphering gene function and developing biotechnology approaches. The ancient TOR-SnRK1 signaling network is an important determinant of yield and broad-spectrum disease resistance in plants, including rice [18,40]. The *ostor, ossnrk1αa*, and *ossnrk1αb + ossnrk1αc* mutants developed in this study are unique resources for genomic, genetic and biochemical investigations on linking this signaling network with growth/development and environmental response in rice. Future studies will be focused on utilizing these genetic resources to understand the role of the rice TOR-SnRK1 signaling network in regulating the growth and environmental responses.

## 4. Materials and Methods

### 4.1. Plant Lines

To develop CRISPR-targeted lines, sgRNA/Cas9 vectors were made in the pRGE32 backbone using the protocol of Xie et al. [41], and the two sgRNA spacer sequences per gene were selected using the CCTop tool [42]. The resulting sgRNA/Cas9 vectors expressed 2 sgRNA for each gene and were named as pNS71 (OsTOR_LOC_Os05g14550), pNS72 (OsSnRK1αB_LOC_Os03g17980+OsSnRK1αC_LOC_Os08g37800), and pNS73 (OsSnRK1αA_LOC_Os05g45420). Each of these vectors was transformed into Oryza sativa var. japonica cv. Kitaake using the gene gun method described earlier using pHPT for hygromycin selection [4]. Briefly, 10 plates containing an approximately equal amount of seed-derived embryogenic callus were co-bombarded with pNS71/72/73 and pHPT, and the hygromycin-resistant callus lines were isolated for plant regeneration. The regenerated plant lines (T0) were transferred to a greenhouse in pots containing a mixture of sphagnum peat moss and perlite (9:1), Sungro SS#1, and Osmocote^®^ fertilizer (15N-9P-12K). Using the same tissue culture, a few non-transformed T0 plants were also regenerated, that were designated as tissue-culture-derived wild-type (WT) lines.

### 4.2. Molecular Analysis

The presence of mutations in each T0 line was determined by PCR and Sanger sequencing. Genomic DNA isolated from the T0 plants was used for the polymerase chain reaction (PCR) using primers spanning the target sites (Table S1). PCR products were resolved on the agarose gel and extracted using the GeneJET Gel Extraction Kit (Thermo Scientific, USA) for sequencing from both ends using the forward and the reverse primers by the Sanger Sequencing method (Psomagen, Rockville, MD, USA). The sequence traces (ABI files) were analyzed on the Sequence Scanner 2 software (Applied Biosystems Inc., Waltham, MA, USA) and aligned with the reference sequences using the CLUSTAL-Omega multiple sequence alignment tool. The overlapping sequences arising from heterozygous alleles were separated using the CRISP-ID or Polypeak Parser tools [43,44].

### 4.3. Phenotypic Analysis

The seeds of wild-type cv. Kitaake and mutant lines were surface sterilized with 30% bleach and grown on ½ Murashige and Skoog media (MS; pH: 5.7) with 20 g/L Sucrose (Sigma-Aldrich, St. Louis, MO, USA) and 2 g/L phytagel (Sigma-Aldrich, St. Louis, MO, USA) in Petri-plates. This composition was used for all experiments until seeds reach the S3 stage, i.e., the emergence of prophyll from coleoptile (3–5 days after plating). For phenotyping *tor* mutants, the S3 stage seedlings were transferred in Borosilicate glass tubes (38 × 200 mm; Bellco Biotechnology) containing ½ MS media supplemented with 8 mM or 60 mM sucrose and solidified with 1.5 g/L phytagel (Sigma-Aldrich, St Louis, MO, USA). The seedlings were cultivated in a growth chamber (Percival-Scientific Inc., Perry, IA, USA) under a 16:8 h photoperiod at 26 °C (day) and 22 °C (night) temperature under a light intensity of 10–30 µmol m^−2^ s^−1^. The shoot length and fresh weight of each seedling were measured 6 days after transferring into the tubes.

For phenotyping *snrk1α* mutants, S3 stage seedlings were transferred to Borosilicate glass tubes containing ½ MS media (without sucrose) solidified with 1.5 g/L phytagel. The seedlings were grown in the growth chamber as described above, except the light intensity was set to maximum (200 µmol m^−2^ s^−1^). Seedlings were photographed 4 days after and seedling length was measured at 6 days after placing the S3 seedlings in the tubes.

Phenotyping in the greenhouse was done using T1 seeds of the mutants along with the wild-type cv. Kitaake. Seeds were surface sterilized and germinated on ½ MS media and 2 weeks old seedlings were transferred to the greenhouse in pots filled with a mixture of sphagnum peat moss and perlite (9:1), Sungro SS#1, and Osmocote^®^ fertilizer (15N-9P-12K). The plants were grown in a randomized block design in a controlled environment in the greenhouse and fertilized with iron chelate and 15N-9P-12K and treated with insecticide as necessary. The plants were harvested 113 days later. The shoot and root lengths were measured with a measurement tape and expressed in centimeters. The panicles, shoots and roots were separated, and the roots were washed in running tap water. Subsequently, plant parts (shoot, roots and panicles) were dried in a 37 °C dryer for 14 days. The dry weight (biomass) of the root and shoot of each plant was recorded and expressed in grams. The number of seeds per panicle was counted manually for each plant.

### 4.4. Statistical Analysis

Phenotypic data were statistically analyzed by analysis of variance (ANOVA), and the Tukey–Kramer test (HSD) for comparisons among treatments using JMP Statistical Discovery from SAS (Version 13.2.1) software.

## Figures and Tables

**Table 1:** Summary of targeting *OsTOR* and *OsSnRK1α* genes.

Gene	CRISPR Construct	No. Of Plates	T0 Lines ^1^	Targeted Lines ^2^	Survivors ^3^	% Transf. ^4^	% Targeting ^5^	Heritable Mutation
*OsTOR*	pNS71	10	21	3	1	52.5	14	In-frame deletion
*OsSnRK1* *αB* *OsSnRK1* *αC*	pNS72	10	10	9	3	25	90	Indels/in-frame deletion
*OsSnRK1* *αA*	pNS73	10	40	30	10	100	75	Indels

^1^ Number of primary transformed lines (T0) recovered from the bombardment of 10 callus plates by the CRISPR construct. ^2^ Number of T0 lines showing targeted mutations. ^3^ Number of fertile mutant lines that transferred the mutations to the progeny. ^4^ Percent recovery of T0 lines from 10 plates containing 4 callus clusters each (40 clusters). ^5^ Number of targeted lines out of a total number of T0 lines × 100.

## Data Availability

Not applicable.

## Supplementary Materials

The following supporting information can be downloaded at: https://www.mdpi.com/article/10.3390/plants11111453/s1, Figure S1: Sequence alignment of predicted TOR protein sequences; Figure S2: Illustration of OsSnRK1α targeting; Figure S3: PCR analysis of pNS73 (OsSnRK1αA) lines. Table S1: PCR primers used in this study.
