# Supplementary material for: Targeting TOR and SnRK1 Genes in Rice with CRISPR/Cas9
Source: Plants (Basel). 2022 May 30;11(11):1453. doi: 10.3390/plants11111453 (PMC9183148; doi:10.3390/plants11111453)
Supplement: Supplementary file 1 [file plants-11-01453-s001.zip › plants-1724489-supplementary.pdf]

Supplementary Information:

Targeting TOR and SnRK1 genes in rice with CRISPR/Cas9

Pathak et al.

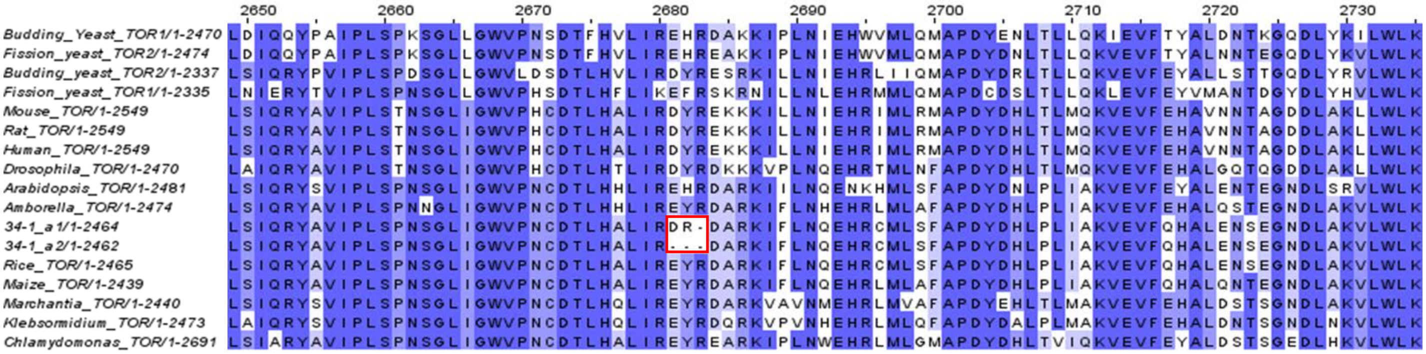

Supplementary Figure S1: Sequence alignment of predicted protein sequences of *ostor* mutant alleles, 34-1_a1 and 34-1_a2, with plant, fungal and animal TOR proteins. The intensity of the blue color indicates the degree of conservation of amino acid residues at a particular position. The sequences were aligned with ClustalX2 and visualized in Jalview (v2.11.2.0). Predicted mutations in 34-1_a1 and 34-1_a2 are boxed with red outline.

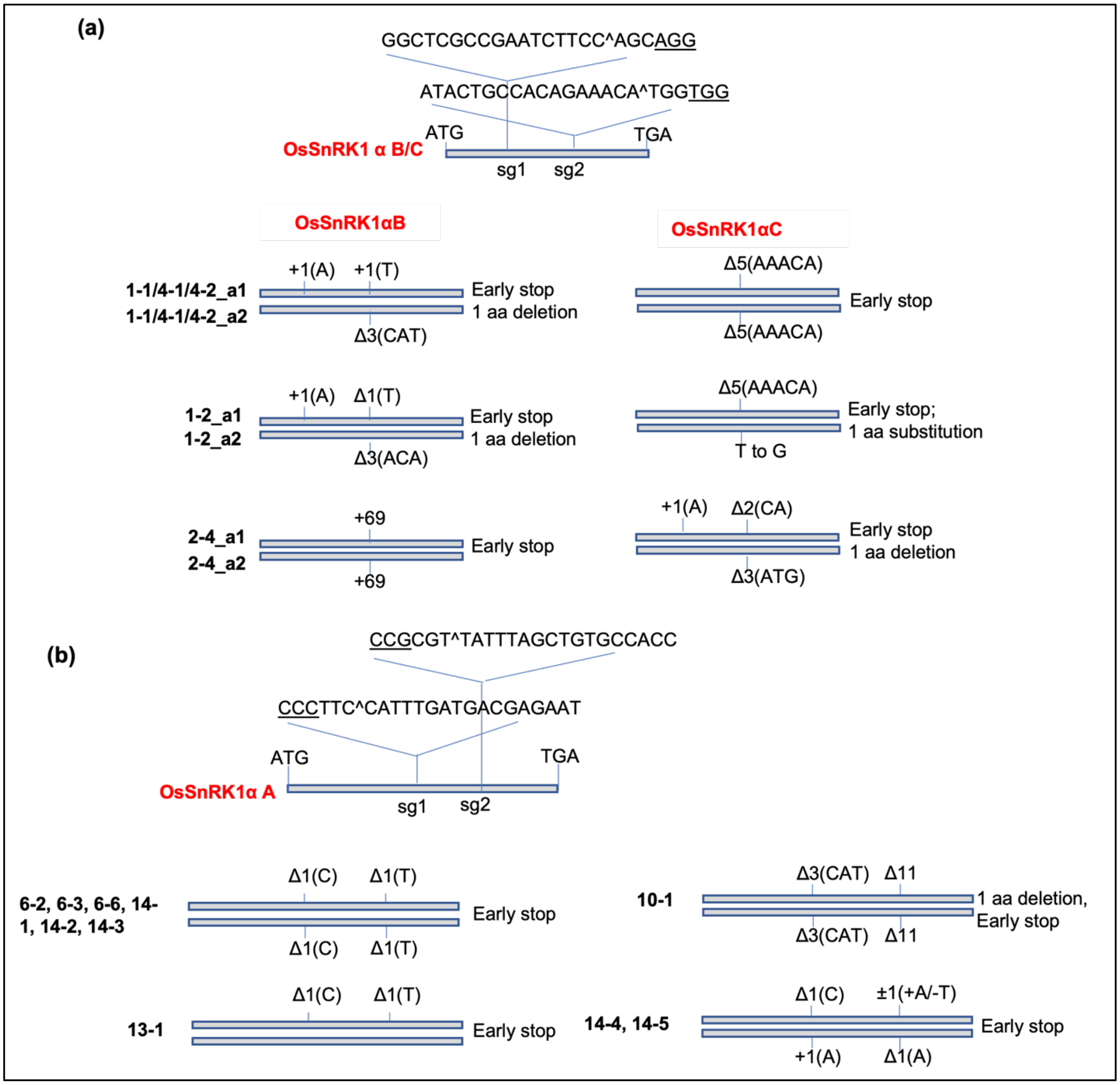

Supplementary Figure S2: Illustration of OsSnRK1α targeting. **(a)** Simultaneous targeting of OsSnRK1αB and OsSnRK1αC at the homologous sites, and **(b)** single targeting of OsSnRK1αA at two sgRNA target sites (sg1 and sg2). Mutations in each allele (a1 and a2) of OsSnRK1αA, OsSnRK1αB, and OsSnRK1αC are shown as insertion (+) or deletions (Δ) and the effect of mutation is indicated. Different T0 lines showing identical mutations are grouped. PAM sequence in each sg site is underlined and the predicted DSB site is indicated (^).

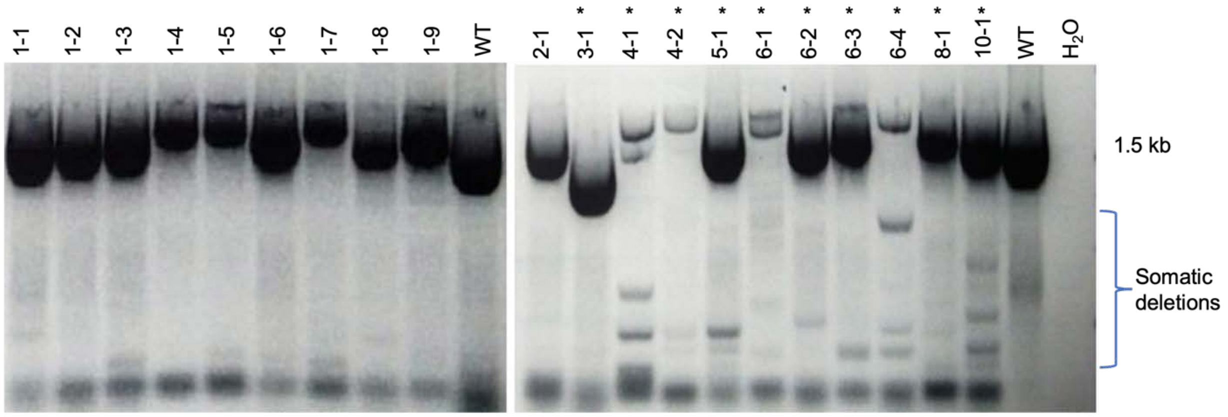

Supplementary Figure S3: PCR analysis of pNS73 (SnRK1αA) lines. PCR was conducted using genomic DNA from T0 lines transformed with pNS73 using primers spanning the sg1 and sg2 target sites and resolved on 0.8% agarose gel. The expected amplicon size is 1.5 kb. Minor bands of smaller than 1.5 kb indicate somatic deletions.

Supplementary Table S1: Primers used in this study

**Gene**	**Primer**	**Primers (5'-3')**	**Application**
OsTOR	TOR-F1	TTATGCAGATTGCTCCTCG	Genotyping of OsTOR target site 1 in the HEAT region
	TOR-R1	TGGATGGCTCAATCAAGG	
	TOR-F2	GGTGAATACTCTGCTGGA	Genotyping of OsTOR target site 2 in the kinase domain
	TOR-R2	CTG ACA CAA GTT TGT TCA GG	
OsSnRK1αA	SnRK1a-F1	ATTGGTCACAGTTGACCAGCC	Genotyping of OsSnRK1αA target sites 1 and 2
	SnRK1a-R3	GAGGAGCAATGGAGTGAAG	
OsSnRK1αB	Sn1ab-F1	TGGAATTTCATGCATGCC	Genotyping of OsSnRK1αB target sites 1 and 2
	Sn1ab-R2	ACCTCTGGAGCAGCATAG	
OsSnRK1αC	Sn1b-F1	CCTGCTAATGCTTGTGAGTG	Genotyping of OsSnRK1αC target sites 1 and 2
	Sn1b-R2	ACCTCTGGAGCAGCATAA	
